# Supplementary material for: Resolving the full spectrum of human genome variation using Linked-Reads
Source: Genome Res. 2019 Apr;29(4):635–45. doi: 10.1101/gr.234443.118 (PMC6442396; doi:10.1101/gr.234443.118)
Supplement: Supplemental Material [file supp_gr.234443.118_Supplemental_longranger-2.2.zip › longranger-2.2.tar.bz2!/lr2.2-public/lib/stan/cmdstan/stan/doc/api/html/elt__multiply_8hpp_source.html]

Stan: src/stan/math/matrix/elt\_multiply.hpp Source File


|  |  |
| --- | --- |
|  | Stan  2.5.0  probability, sampling & optimization |


- Main Page
- Related Pages
- Namespaces
- Classes
- Files

- File List
- File Members

All Classes Namespaces Files Functions Variables Typedefs Enumerations Enumerator Friends Macros Pages

- src
- stan
- math
- matrix

elt\_multiply.hpp

Go to the documentation of this file.

1 #ifndef STAN\_\_MATH\_\_MATRIX\_\_ELT\_MULTIPLY\_HPP

2 #define STAN\_\_MATH\_\_MATRIX\_\_ELT\_MULTIPLY\_HPP

3

4 #include <boost/math/tools/promotion.hpp>

5 #include <stan/math/matrix/Eigen.hpp>

6 #include <stan/math/error\_handling/matrix/check\_matching\_dims.hpp>

7

8 namespace stan {

9  namespace math {

10

23  template <typename T1, typename T2, int R, int C>

24  Eigen::Matrix<typename boost::math::tools::promote\_args<T1,T2>::type, R, C>

25  elt\_multiply(const Eigen::Matrix<T1,R,C>& m1,

26  const Eigen::Matrix<T2,R,C>& m2) {

27  stan::math::check\_matching\_dims("elt\_multiply(%1%)",m1,"m1",

28  m2,"m2",(double\*)0);

29  Eigen::Matrix<typename boost::math::tools::promote\_args<T1,T2>::type, R, C>

30  result(m1.rows(),m2.cols());

31  for (int i = 0; i < m1.size(); ++i)

32  result(i) = m1(i) \* m2(i);

33  return result;

34  }

35

36  }

37 }

38 #endif

check\_matching\_dims.hpp

stan::math::check\_matching\_dims

bool check\_matching\_dims(const char \*function, const Eigen::Matrix< T1, R1, C1 > &y1, const char \*name1, const Eigen::Matrix< T2, R2, C2 > &y2, const char \*name2, T\_result \*result)

**Definition:** check\_matching\_dims.hpp:15

Eigen.hpp

stan::math::elt\_multiply

Eigen::Matrix< typename boost::math::tools::promote\_args< T1, T2 >::type, R, C > elt\_multiply(const Eigen::Matrix< T1, R, C > &m1, const Eigen::Matrix< T2, R, C > &m2)

Return the elementwise multiplication of the specified matrices.

**Definition:** elt\_multiply.hpp:25


---

[ Stan Home Page ]

*© 2011–2014,
Stan Development Team.*
